# Winter diet of Japanese macaques from Chubu Sangaku National Park, Japan incorporates freshwater biota

**DOI:** 10.1038/s41598-021-01972-2

**Published:** 2021-11-29

**Authors:** Alexander M. Milner, Susanna A. Wood, Catherine Docherty, Laura Biessy, Masaki Takenaka, Koji Tojo

**Affiliations:** 1 School of Geography, Earth and Environmental Science, University of Birmingham, Birmingham, UK; 2 Department of Biology, Faculty of Science, Shinshu University, Matsumoto, Japan; 3 Cawthron Institute, Nelson, New Zealand; 4 Sugadaira Research Station, Mountain Science Center, University of Tsukuba, Ueda, Japan; 5 Institute of Mountain Science, Shinshu University, Matsumoto, Japan

**Keywords:** Ecology, Limnology

## Abstract

The Japanese macaque (*Macaca fuscata*) is native to the main islands of Japan, except Hokkaido, and is the most northerly living non-human primate. In the Chubu Sangaku National Park of the Japanese Alps, macaques live in one of the coldest areas of the world, with snow cover limiting the availability of preferred food sources. Winter is typically a bottleneck for food availability potentially resulting in marked energy deficits, and mortality may result from famine. However, streams with groundwater upwelling flow during the winter with a constant water temperature of about 5 °C are easily accessible for Japanese macaques to search for riverine biota. We used metabarcoding (Cytochrome c oxidase I) of fecal samples from Japanese macaques to determine their wintertime diet. Here we provide the first robust evidence that Japanese macaques feed on freshwater biota, including brown trout, riverine insects and molluscs, in Chubu Sangaku National Park. These additional food sources likely aid their winter survival.

## Introduction

Japanese macaques (*Macaca fuscata*) typically feed on fruits, seeds, mature and young leaves, and flowers, but are also known to consume fungi and terrestrial invertebrates^1,2^. The fruits available to macaques have been shown to be affected by habitats changing close to streams^3^. A diverse number of terrestrial invertebrate taxa are also eaten by Japanese macaques^1,4^. Despite their small size invertebrates provide larger amounts of energy, protein and fat per unit mass than other foods, such as fruit and vegetation^5^. In winter their preferred foods are not widely available in snowy areas, so Japanese macaques typically consume the bark and buds of woody plants^6–8^, particularly in areas of Japan, like Kamikochi and Shiga Heights^9–12^. The diet of Japanese macaques in heavy snow conditions is typically influenced more by resource availability than by diet preference^12^. Although, invertebrates may represent an alternative food source in winter, typically feeding on terrestrial insects by Japanese macaques is strongly correlated with higher air temperature as the availability of insects increases from June to August^13,14^. With snow cover inhibiting their preferred food sources during the winter, groundwater influenced streams with no ice cover are easily accessible for Japanese macaques to turn over cobbles and potentially feed on riverine biota. Macaques in Kamikochi have been circumstantially documented to potentially eat aquatic insects during the winter season through binocular observations^15^. However, from these observations it was not known which species were consumed as some of the taxa documented in the stream are not found in Kamikochi. In this study we used metabarcoding of the Cytochrome c oxidase I (COI) gene to examine fecal samples from Japanese macaques in the mountainous area of Kamikochi in Chubu Sangaku National Park to determine their wintertime diet conclusively. The samples were collected over three separate winter sampling periods.

## Results

A total of 1,896 Amplicon Sequence Variants (ASVs) were found from the 38 samples of Japanese macaque feces collected in the winter seasons 2017 to 2019 (see Materials and Methods). To explore the component of the diet sourced from the streams, we then focused only on sequences from freshwater organisms. DNA from fish were present in seven fecal samples, molluscs in nine samples and arthropods with an aquatic life stage in 18 samples (Table 1).

**Table 1:**
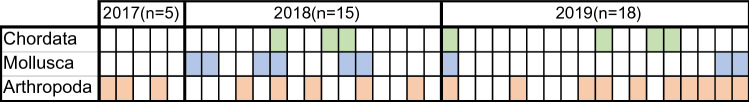
Presence (coloured) or absence (white) of freshwater organisms (at the order level) in the fecal samples of Japanese macaques collected near the Azusa River in Kamikochi (Chubu Sangaku National Park), Japan.

When looking at the entire eukaryotic community, the dominant phylum in the *M. fuscata*’s feces in 2017 and 2019 was Arthropoda (16–100% relative abundance in each sample) while Chordata was dominant in 2018 (0–100%). Molluscs were also abundant in the feces in 2018 and 2019, but only in nine samples and at a lower relative abundance (2–61%).

The freshwater fish *Salmo trutta* (brown trout) was definitively identified in 2018 and 2019 from the fecal samples (Table 2). The sequences had 100% sequence similarity to other *Salmo trutta* entries in the genetic sequence database GenBank (https://www.ncbi.nlm.nih.gov/genbank/). The freshwater mollusc species found in the diet (*Potamopyrgus antipodarum*, the New Zealand mud snail, and *Semisulcospira dolorosa*) were identified with > 99% sequence similarity in 2018 and 2019 but were not present in 2017. Freshwater arthropod insect sequences were found in all years with five genera detected. Two genera were Plecoptera, *Nemoura fluva* and *Sweltsa* sp*.,* the other three were *Conchapelopia* sp. (Chironomidae), *Tipula* sp. (Tipulidae) and *Dixa* sp. (Dixidae). *Nemoura fluva* showed over 98% sequence similarity and the other four genera over 90% similarity to sequences in GenBank. One species of freshwater copepod was also found, *Mesocyclops leuckrati,* with over 99% sequence similarity.

**Table 2:** BLAST results from freshwater chordates, molluscs and aquatic arthropods (or arthropods having aquatic life stages) sequences identified in the fecal samples of Japanese macaques.

Phylum	Class	Order	Family	Genus/species	% similarity	% coverage	Number of reads
Chordata	Actinopterygii	Salmoniformes	Salmonidae	*Salmo trutta*	100	100	2,745
Mollusca	Gastropoda	Littorinimorpha	Tateidae	*Potamopyrgus antipodarum*	99.7	100	16,103
	Gastropoda	Pleuroceridae	Semisulcospiridae	*Semisulcospira dolorosa*	100	100	179
Arthropoda	Hexanauplia	Cyclopoida	Cyclopidae	*Mesocyclops leuckarti*	99.7	100	6,842
	Insecta	Plecoptera	Nemouridae	*Nemoura fulva*	98.7	100	6
	Insecta	Diptera	Tipulidae	*Tipula* sp.	94.9	100	440
	Insecta	Diptera	Chironomidae	*Conchapelopia* sp.	93.2	100	203
	Insecta	Plecoptera	Chloroperlidae	*Sweltsa* sp.	90.6	100	61
	Insecta	Diptera	Dixidae	*Dixa* sp.	90.4	100	6
	Insecta	Diptera	Chironomidae		87.5	100	2,547

% similarity = percentage of similarity between the sequences found in the fecal samples and the sequences in the GenBank database; % coverage = percentage of the sequence found in our sample aligned to a sequence in GenBank; number of reads = number of times that same sequence occurred in the samples.

## Discussion

To the best of our knowledge, there are no previous published accounts of Japanese macaques definitively eating freshwater animals in streams, including brown trout. Feeding strategies of Japanese macaques change according to seasonal fluctuations in food resources^16,17^ and typically winter is a bottleneck for food availability^18^ thereby potentially resulting in a significant energy deficit^19^. Japanese macaques are at their upper elevation limit (1500 m) in Kamikochi and mortality may result from famine or hypothermia which tends to occur towards the end of winter in March and April^20^.

Consumption of vertebrates is rare^21^. Previously, Japanese macaques have been shown to opportunistically capture marine fish, either when being dried^22^ or washed up on beaches^23^. Closely related species have been shown to feed on freshwater fish. For example, long tailed macaques actively capture fish from freshwater pools^24^, and wild chacma baboons from drying desert pools^25^. Presumably, the Japanese macaques follow a similar approach capturing brown trout in shallow pools along the stream margin although they may opportunistically eat dead fish although none were observed despite extensive wintertime sampling of the benthos. The capture of live fish has been suggested as probably rare in Japanese macaques^25^ but brown trout were found in seven fecal samples from 2018 and 2019. Unfortunately, using metabarcoding, it is not possible to determine, whether the DNA signal came from different individual fish in each subsequent year.

Animals attached to rocks require less energy expenditure to capture than fish. Although terrestrial insects are a major source of food for Japanese macaques, especially in summer, this is the first confirmed record of aquatic insect larvae and nymphs in their diet. Of the five genera that were found, three (*N. fluva* and *Sweltsa* sp.*,* the two stoneflies, and the dipteran *Tipula* sp.) are relatively large (>1 cm) at the final instar stage*.* At the time of year the fecal samples were collected, these insects would not be present as adults, in their terrestrial life-cycle stage, so we conclude that they were captured from the freshwater environment. Japanese macaques collecting insects by turning stones over in the stream is a form of extractive foraging^26^.

Japanese macaques have previously been reported to eat marine mussels^27^ and terrestrial snails and slugs^11,28^. The Burmese long-tailed macaque had been shown to use stone tools to crack open shellfish and this foraging task is time intensive^29,30^. One of the snails *Potamopyrgus antipodarum*, the New Zealand mud snail eaten at Kamikochi, is an invasive species first identified in Japan in 1990^31,32^ with an average shell size of 0.5 cm. These molluscs are easily removed from the stones and we assume they are swallowed whole and feeding on them does not involve forceful extraction from the shell, as with marine mussels.

Due to their relatively small size, we speculate that the freshwater copepod, *Mesocyclops leuckrati* was consumed through the Japanese macaques drinking stream water.

**Figure 1:**
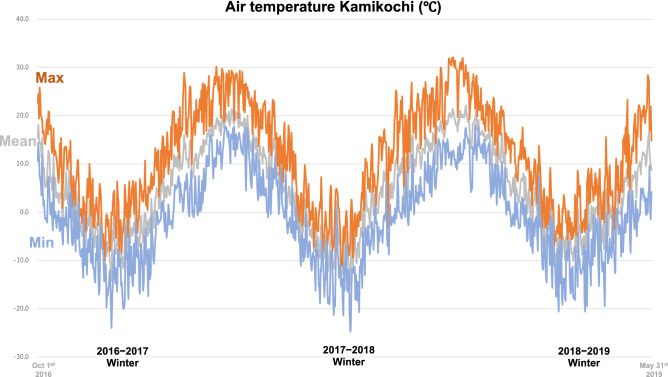
Winter air temperature at the Kamikochi study site during the period 2017 to 2019.

In general, Japanese macaques have a wider home range in winter when food resources are scarce. However, the Kamikochi area lies in a deep valley where the surrounding mountain ranges are >2500 m and have steep inclines, so their home range cannot be expanded. As a result, the population density is exceptionally high, increasing from 90 individuals in two troops in 1990 to 205 individuals in four troops in 2018^6,33^. These Kamikochi macaques are thus forced to overwinter within an extremely harsh environment with high snowfall (mean max depth for 2017–2019 was 930 mm) and low winter temperature (minimums to –20 °C see Fig. 1). These larger populations create additional stress for overwintering survival. An abundance of groundwater upwellings and hot spring inputs from active volcanoes in the Kamikochi region ensures many streams are flowing over the winter without ice cover allowing easy access to the monkeys. Many streams have a stable water temperature year-round (5 to 6 °C) contributing to high biomass of fish and freshwater benthos^34^ which would be accessible to the Japanese macaques. Hence, the Kamikochi area may be the only environment in Japan where the topographical, geological, and meteorological conditions facilitate this unique feeding of freshwater biota by Japanese macaques to supplement their winter diet.

## Methods

Samples were collected by locating fresh feces close to the Azusa River in Kamikochi (Chubu Sangaku National Park 36°15′09.80"N 137°40′02.33"E). Small amounts (ca. 1 g) were sampled from the centre of the feces with a sterilized spatula and placed in a 15 ml tube containing LifeGuard Soil Preservation Solution (QIAGEN, CA, USA). Five samples were collected December 2017, 15 samples in February 2018, and 18 samples in late March/early April 2019. These samples were at least 50 m apart to minimise the chance that fecal samples were from the same individual and in 2018 and 2019 samples were collected at least 7 days apart.

Each step of the molecular analysis (DNA extraction, PCR setup, template addition, electrophoresis and PCR clean-up) was conducted in a separate sterile laboratory dedicated to that step with sequential workflow to ensure no contamination. Each room was equipped with ultra-violet sterilization which was switched on for a minimum of 15 min before and after each use. The PCR set-up and template addition were undertaken in laminar flow cabinets with HEPA filtration.

Each sample was homogenized using sterile beads (2 min, 1,500 RPM; 1600 MiniG Automated Tissue Homogenizer and Cell Lyser, SPEX SamplePrep, NJ, USA) and a subsample (ca. 0.2 g) weighted directly into the first tube of a DNeasy PowerSoil DNA Isolation Kit (QIAGEN, CA, USA). DNA was extracted following manufacturer’s instructions using a robotic workstation (QIAcube, QIAGEN). Samples were frozen (−20 °C) until further processing and negative extraction controls were included in each run to monitor contamination.

As Japanese macaque DNA was potentially more prevalent than dietary organisms during high-throughput sequencing (HTS), a blocking primer approach was used to inhibit DNA amplification of the Japanese macaque during the PCR step^35^. A blocking primer was designed to overlap with the 3′-end of the forward PCR primer and modified with a C3 spacer. The blocking primer was included at 10 times the concentration of PCR primers during amplification.

For metabarcoding, a segment of approximately 300 bp of the mitochondrial cytochrome oxidase 1 (COI) gene was amplified by PCR using the primers mlCOIintF: 5’-GGW ACW GGW TGA ACW GTW TAY CCY CC-3’ and jgHCO2198: 5’-TAI ACY TCI GGR TGI CCR AAR AAY CA-3′^36^ and the blocking primer *M. fuscata*-blocker 5’-ATA TTC CCCCCT AGC AGG AAA CTT CTC C /3SpC3/-3’ designed in this study. PCR amplification was undertaken in a total volume of 50 μL using 25 μL of MyFi™ DNA Polymerase (Bioline, USA), 1 μL of each primer (10 µM), 1 μL of blocking primer (100 µM), 15 µL of DNA-free water, 2 µL of BSA (0.2 mg mL^−1^; Sigma, USA) and 5 µL of template DNA (ca. 5 ng µL^−1^). Thermocycling conditions were: 95 °C for 5 min, followed by 40 cycles of 95 °C for 20 s, 52 °C for 20 s, 72 °C for 60 s, and a final extension of 72 °C for 10 min. Two samples of RNA/DNA-free water were used as negative controls following the protocol described above.

PCR products were visualized on 1.5% agarose gel with Red Safe™ DNA Loading Dye (Herogen Biotech, USA) and UV illumination. PCR negatives were run to assess for contamination during the PCR steps. The PCR products were purified, cleaned of primer dimers and normalized using SequalPrep Normalisation plate (ThermoFisher, MA, USA), and submitted to Auckland Genomics (University of Auckland, New Zealand) for library preparation. Sequencing adapters and sample-specific indices were added to each amplicon via a second round of PCR using the Nextera™ Index kit (Illumina Inc., USA). Quality control was undertaken using a bioanalyzer before the library was diluted to 4 nM and denatured. A 15% PhiX spike was used and the final loading concentration was 7 rM. Sequence data were automatically demultiplexed using MiSeq® Reporter (version 2, Illumina Inc.), and forward and reverse reads assigned to samples. Raw sequence reads were deposited in the National Center for Biotechnology Information (NCBI) short read archive under the accession number PRJNA706701.

Raw reads were processed, subsequent to primers being removed with *cutadapt*^37^ using the *DADA2* package^38^ within R. Reads were truncated to 221 and 223 bp and filtered with a maxEE (maximum number of “expected errors”) of 2 and 2 for forward and reverse reads respectively (reads not reaching this threshold were discarded). *DADA2* constructs a parametric error matrix (based on the first 10^8^ bps in the dataset), the samples are dereplicated and sequence variants for the forward and reverse reads are inferred based on the derived error profiles from the samples. Singletons observed in the inference step are discarded. Subsequently, paired-end reads were merged with a maximum mismatch of 1 bp and a required minimum overlap of 10 bp. Forward and reverse reads, which did not merge were not included in further analysis. Chimeras were removed using the function removeBimeraDenovo. The resulting chimera-checked, merged Amplicon Sequence Variants (ASVs) were used for taxonomic classification using a redundant BOLD database^39^ supplemented with COI sequences from NCBI within the *DADA2* package, which is based on the rdp classifier^40^ with a bootstrap of 50. The results were parsed into a table using the *phyloseq* package^41^, and reads assigned as primates were removed. The differentiation of the sequences from the annelids present in the Japanese macaque’s intestinal tract already from those in the diet was not feasible and thus removed. Negative controls were assessed and the sum of reads from contaminating ASVs was subtracted from the samples.

Phylogenetically annotated COI sequences were used to characterize eukaryotic composition for each fecal sample using the package *ggplot2*^42^ in R. Sequences of interest (freshwater arthropods, molluscs and fish) were selected and blasted using the BOLD and the NCBI databases for a more accurate taxonomy identification.

## Data availability

Raw sequence reads were deposited in the National Center for Biotechnology Information (NCBI) short read archive under the accession number PRJNA706701.

## Supplementary Information

Supplementary Information.
