# Supplementary material for: Winter diet of Japanese macaques from Chubu Sangaku National Park, Japan incorporates freshwater biota
Source: Sci Rep. 2021 Nov 29;11:23091. doi: 10.1038/s41598-021-01972-2 (PMC8629975; doi:10.1038/s41598-021-01972-2)

Supplementary Figure 1. Stacked bar plot showing the relative abundance of mitochondrial cytochrome oxidase subunit sequences at the phylum level for only organisms identified as having a freshwater habitat in the feces samples of the Japanese Macaque. Note non-freshwater taxa were identified but these are not displayed.


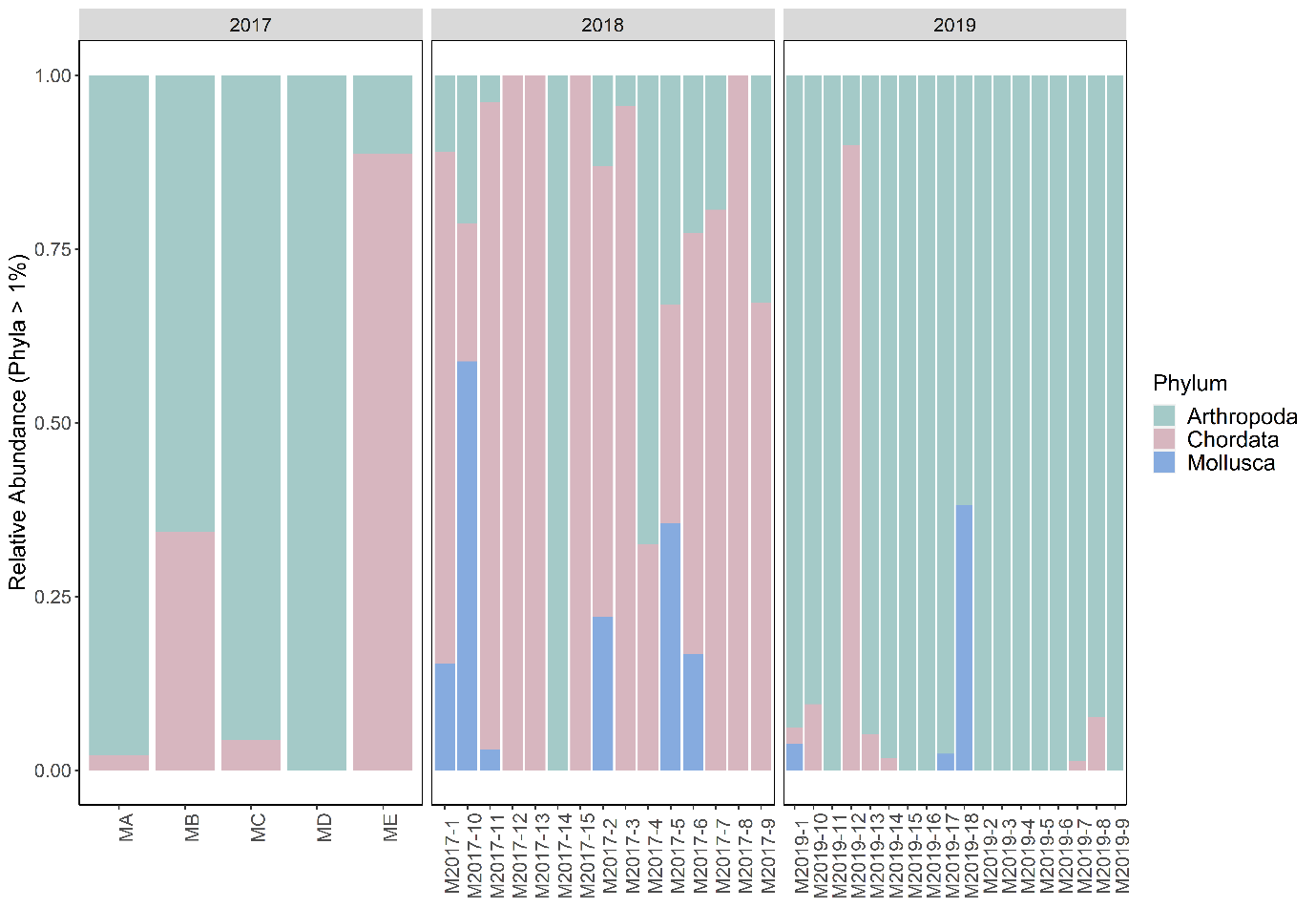

Supplement: Supplementary file 1 — Supplementary Information. [file 41598_2021_1972_MOESM1_ESM.docx]
